# Differential Regulation of Myocardial E3 Ligases and Deubiquitinases in Ischemic Heart Failure

**DOI:** 10.3390/life11121430

**Published:** 2021-12-18

**Authors:** Kristin Klaeske, Maria Dix, Volker Adams, Khalil Jawad, Sandra Eifert, Christian Etz, Diyar Saeed, Michael A. Borger, Maja-Theresa Dieterlen

**Affiliations:** 1 Department for Cardiac Surgery, HELIOS Clinic, Heart Center, University Hospital Leipzig, Strümpellstraße 39, 04289 Leipzig, Germany; Maria.Dix@helios-gesundheit.de (M.D.); Khalil.Jawad@medizin.uni-leipzig.de (K.J.); sandra.eifert@helios-gesundheit.de (S.E.); christian.etz@helios-gesundheit.de (C.E.); Diyar.Saeed@helios-gesundheit.de (D.S.); Michael.Borger@helios-gesundheit.de (M.A.B.); Maja-Theresa.Dieterlen@helios-gesundheit.de (M.-T.D.); 2 Laboratory of Molecular and Experimental Cardiology, Heart Center Dresden, TU Dresden, Fetscherstraße 76, 01307 Dresden, Germany; volker.adams@tu-dresden.de; 3 Dresden Cardiovascular Research Institute and Core Laboratories GmbH, Bautzner Straße 122c, 01099 Dresden, Germany

**Keywords:** E3 ligases, deubiquitinase, myocardial infarction, chronic heart failure

## Abstract

The pathological changes of ubiquitination and deubiquitination following myocardial infarction (MI) and chronic heart failure (CHF) have been sparsely examined. We investigated the expression of muscle-specific E3 ubiquitin ligases and deubiquitinases in MI and CHF. Therefore, mice were assigned to coronary artery ligation for 3 days or 10 weeks as well as for sham operation (each *n* = 10). Expression of E3 ligases (MAFBX, MURF1, CHIP, ITCH, MDM2) and deubiquitinases (A20, CYLD, UCH-L1, USP14, USP19) was determined. After MI and in CHF, the mRNA expression of MURF1, CHIP and MDM2 (all *p* < 0.05) was decreased. Protein expression analyses revealed that ITCH expression decreased in CHF (*p* = 0.01), whereas MDM2 expression increased in MI (*p* = 0.02) and decreased in CHF (*p* = 0.02). Except for USP19 mRNA expression that decreased at 3 days and 10 weeks (both *p* < 0.01), the expression of other deubiquitinases remained unaffected after MI and CHF. The expression of myocardial E3 ligases is differentially regulated following MI, raising the question of whether an upstream regulation exists that is activated by MI for tissue protection or whether the downregulation of E3 ligases enables myocardial hypertrophy following MI.

## 1. Introduction

The ubiquitin proteasome system (UPS) is the main cellular system regulating intracellular protein turnover [1]. In the UPS, ubiquitination and deubiquitination are two contrary events controlling the degradation and stabilization of most cellular proteins [2]. Although ubiquitination requires an enzymatic cascade of ubiquitin-transferring proteins, namely E1 (ubiquitin-activating enzyme), E2 (ubiquitin-transferring enzyme) and E3 (ubiquitin ligase), deubiquitination is mediated by a class of proteins called deubiquitinases (DUBs) [3]. DUBs regulate ubiquitin modifications by binding to a protein substrate that deubiquitinates or by directly binding to a ubiquitin signal that they cleave [4].

Alterations such as the accumulation of ubiquitinated proteins, the proteasome activity and the expression of E2 and E3 enzymes in the UPS have been reported in cardiac diseases [5,6]. Several studies have suggested a key role of E3 ligases in the regulation of myocardial hypertrophy [1,3,5,6]. However, the pathological changes related to ubiquitinating and deubiquitinating enzymes following myocardial infarction (MI) and chronic heart failure (CHF) have been sparsely examined. Therefore, we used a rodent model of MI and CHF to investigate the changes in the expression of muscle-specific E3 ligases and DUBs induced by ischemic cardiac damage. This study included the analysis of the E3 ligases muscle RING finger protein-1 (MURF1), muscle atrophy F-box protein (MAFBX), itchy E3 ubiquitin protein ligase (ITCH), carboxyl terminus of Hsp70-interacting protein (CHIP), mouse double minute 2 homolog (MDM2), and the DUBs ubiquitin-specific protease (USP)14, USP19, TNF-α-induced protein 3 (A20), ubiquitin carboxy-terminal hydrolase L1 (UCH-L1) and CYLD lysine 63 deubiquitinase (CYLD). The present study aimed to assess the expression of myocardial E3 ligases and DUBs following MI to determine the changes associated with myocardial remodelling.

## 2. Results

### 2.1. Animal Characteristics

Three days after left anterior descending (LAD) coronary artery ligation, the mice showed an increase in heart weight (*p* < 0.01) and a reduction in body weight (*p* < 0.01) (Table 1). Echocardiography confirmed reduction in the ejection fraction and fractional shortening (both *p* < 0.01) in the mice. Histological analysis using the hematoxylin and eosin (HE) staining method confirmed the infarct size following MI (57.1 ± 3.3%) and in CHF (42.5 ± 13.1%). Representative examples of HE-stained sections from a sham and a MI mouse were shown previously [7].

The body weights of the mice were found to be similar between the test and the control groups (*p* = 0.90), after 10 w of LAD coronary artery ligation and the sham operation, respectively. In the CHF group, an increase in the heart weight (*p* = 0.02) and a decrease in the ejection fraction and fractional shortening (both *p* < 0.01) were observed as compared with the 10 w control. The infarct size was estimated to be 42.5 ± 13.1% in the CHF group.

### 2.2. mRNA and Protein Expression of E3 Ligases

**Figure 1:**
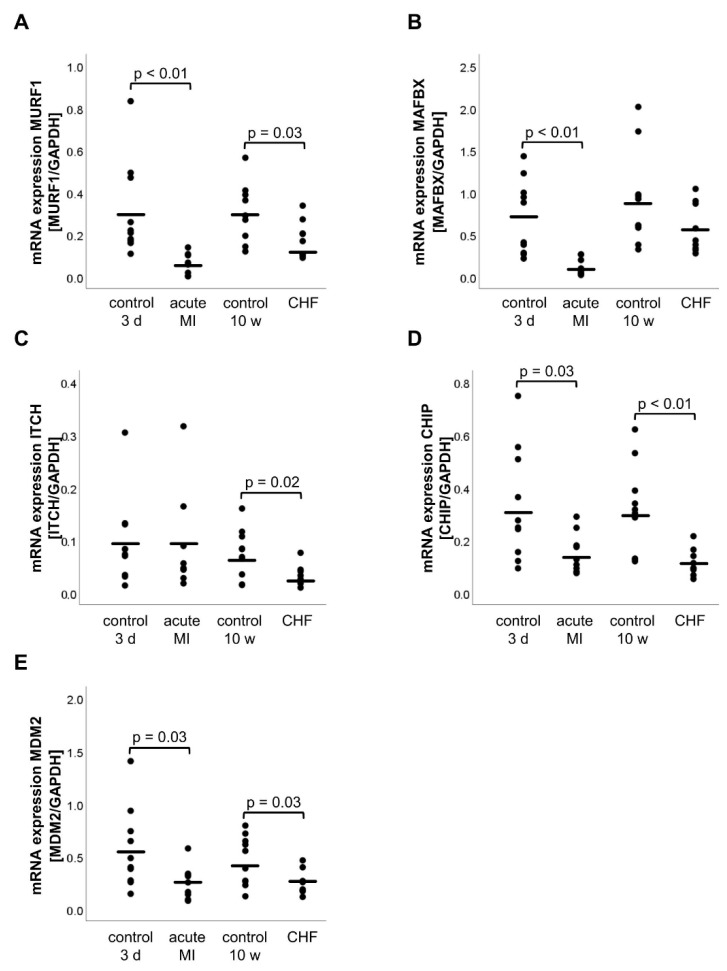
mRNA expression of the E3 ligases MURF1 (**A**), MAFBX (**B**), ITCH (**C**), CHIP (**D**) and MDM2 (**E**). Relative gene expression was determined by RT-qPCR, normalized to the housekeeping gene GAPDH and calculated by the 2^−ΔΔCT^ method. Statistically significant results (*p* ≤ 0.05) were indicated. CHF, chronic heart failure; CHIP, carboxyl-terminus of Hsc70 interacting protein; GAPDH, glyceraldehyde 3-phosphate dehydrogenase; ITCH, E3 ubiquitin-protein ligase Itchy homolog; MAFBX, muscle atrophy F-box; MDM2, mouse double minute 2 homolog; MI, myocardial infarction; mRNA, messenger ribonucleic acid; MURF1, muscle ring finger 1; RT-qPCR, real-time quantitative polymerase chain reaction; 3 d, 3 days; 10 w, 10 weeks.

Compared with the 3 d control group, the mRNA expression of the E3 ligases decreased significantly in the acute MI group (Figure 1) MURF1 (acute MI: 0.07 ± 0.02, 3 d control: 0.31 ± 0.71, *p* < 0.01), MAFBX (acute MI: 0.12 ± 0.03, 3 d control: 0.70 ± 0.14, *p* < 0.01), CHIP (acute MI: 0.15 ± 0.25, 3 d control: 0.33 ± 0.67, *p* = 0.03) and MDM2 (acute MI: 0.25 ± 0.05, 3 d control: 0.57 ± 0.12, *p* = 0.03) except ITCH (acute MI: 0.10 ± 0.32, 3 d control: 0.10 ± 0.26, *p* = 0.89). In the CHF group, a significant decrease in the mRNA expression was observed for MURF1 (CHF: 0.18 ± 0.09, 10 w control: 0.30 ± 0.13, *p* = 0.03), ITCH (CHF: 0.03 ± 0.02, 10 w control: 0.07 ± 0.05, *p* = 0.02), CHIP (CHF: 0.11 ± 0.17, 10 w control: 0.32 ± 0.53, *p* < 0.01) and MDM2 (CHF: 0.26 ± 0.11, 10 w control: 0.46 ± 0.23, *p* = 0.03) (Figure 1). However, the mRNA expression of the E3 ligase MAFBX (*p* = 0.12) did not change significantly in the CHF group compared with the 10 w control.

**Figure 2:**
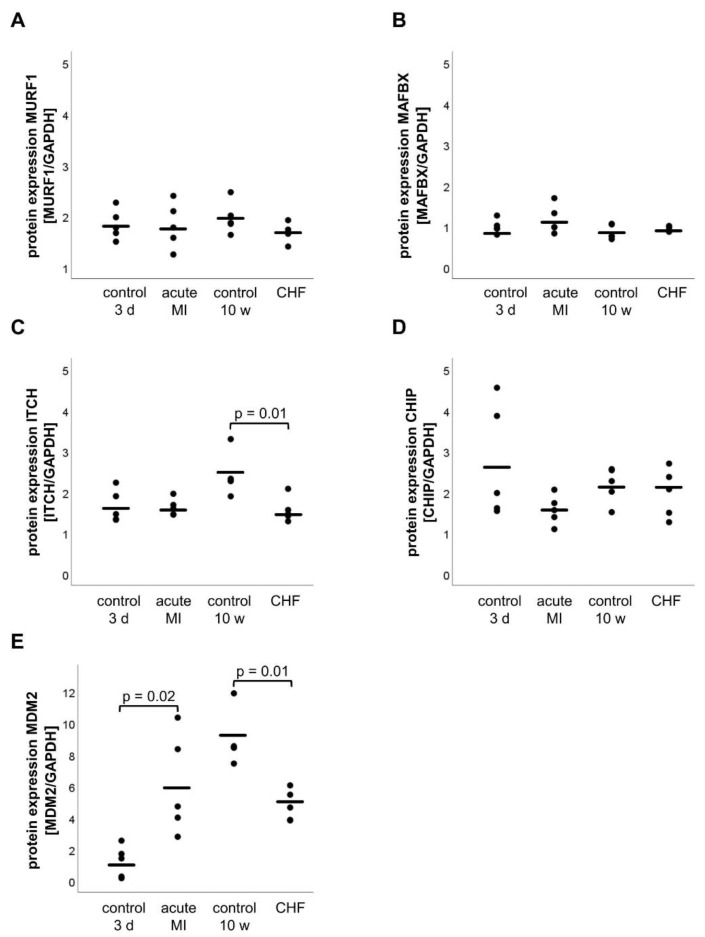
Protein expression of the E3 ligases MURF1 (**A**), MAFBX (**B**), ITCH (**C**), CHIP (**D**) and MDM2 (**E**). Protein expression was determined by Western blot analysis and normalized to GAPDH protein expression. Statistically significant results (*p* ≤ 0.05) were indicated.

**Figure 3:**
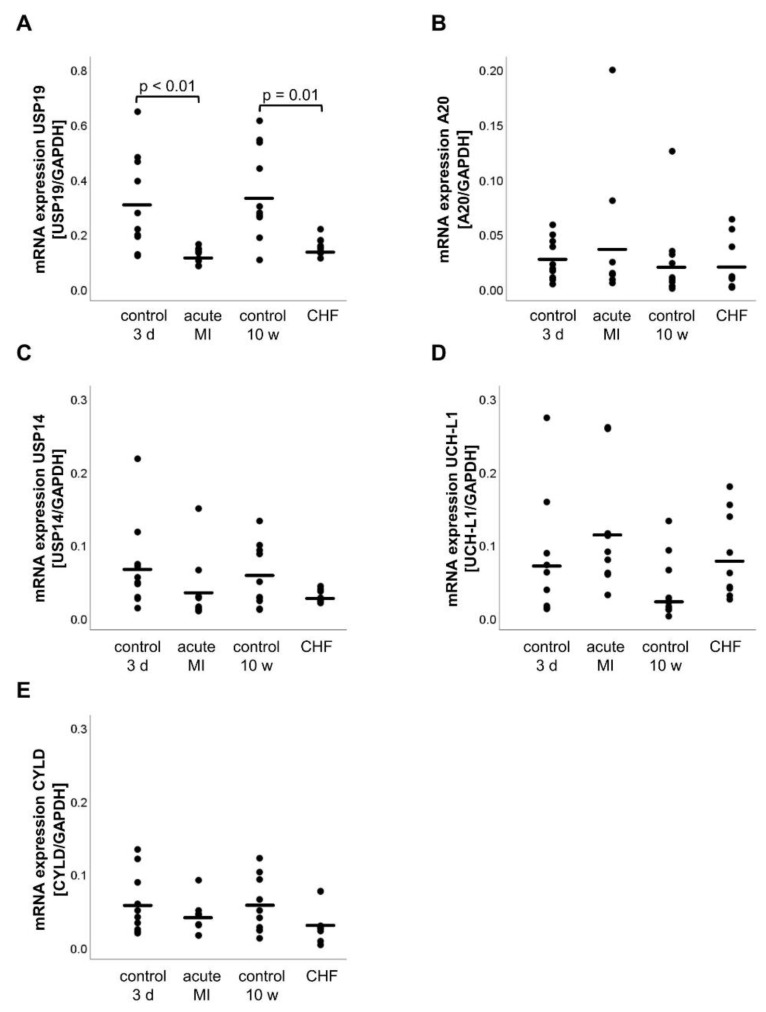
mRNA expression of the deubiquitinases USP19 (**A**), A20 (**B**), USP14 (**C**), UCH-L1 (**D**) and CYLD (**E**). Relative gene expression was determined by RT-qPCR, normalized to the housekeeping gene GAPDH and calculated by the 2^−ΔΔCT^ method. Statistically significant results (*p* ≤ 0.05) were indicated. A20, TNF-α-induced protein 3; CYLD, CYLD lysine 63 deubiquitinase; UCH-L1, ubiquitin carboxy-terminal hydrolase L1; USP14/19, ubiquitin carboxyl-terminal hydrolase 14/19.

In addition to the mRNA expression analysis, protein expression of the E3 ligases was investigated via Western blotting (Figure S1 in supplementary). Neither in the acute phase of MI nor in CHF, a reduction in protein expression could be detected for any of the E3 ligases when compared with their control groups MURF1 (acute MI: 1.82 ± 0.20 arb. U, 3 d control: 1.84 ± 0.13 arb. U, *p* = 0.94; CHF: 1.68 ± 0.08 arb. U, 10 w control: 1.97 ± 0.14 arb. U, *p* = 0.51), MAFBX (acute MI: 1.16 ± 0.16 arb. U, 3 d control: 0.10 ± 0.08 arb. U *p* = 0.37; CHF: 0.93 ± 0.03 arb. U, 10 w control: 0.86 ± 0.08 arb. U, *p* = 0.43) and CHIP (acute MI: 1.57 ± 0.16 arb. U, 3 d control: 2.71 ± 0.63 arb. U, *p* = 0.14; CHF: 2.18 ± 0.19 arb. U, 10 w control: 2.18 ± 0.19 arb. U, *p* = 0.57) (Figure 2A–D). However, ITCH and MDM2 seemed to play an important role in MI and CHF because ITCH protein expression decreased in CHF (CHF: 1.56 ± 0.14 arb. U, 10 w control: 2.41 ± 0.23 arb. U, *p* = 0.01), whereas MDM2 protein expression increased in acute MI (acute MI: 6.05 ± 1.42 arb. U, 3 d control: 1.22 ± 1.01 arb. U, *p* = 0.02) and decreased in CHF (CHF: 4.78 ± 0.44 arb. U, 10 w control: 8.96 ± 1.70 arb. U, *p* = 0.02) compared with the control groups (Figure 2C and Figure 3E). It should be mentioned that the expression of MDM2 increased significantly from 1.22 ± 0.45 arb. U (3 d control = 12 weeks old mice) to 8.96 ± 0.76 arb. U (10 w control = 22 weeks old mice) (*p* < 0.01) with age.

### 2.3. mRNA Expression of the CHIP-Interacting Molecule BAG3 (Bcl-2-Associated Athanogene 3)

The mRNA expression of BAG3 was significantly reduced in the acute MI group (0.23 ± 0.04, *p* = 0.02) as compared with the 3 d control (0.59 ± 0.12). However, in the CHF group (0.53 ± 0.10), BAG3 mRNA expression was comparable with the control group (0.92 ± 0.16, *p* = 0.07).

### 2.4. mRNA Expression of DUBs

The DUB mRNA analysis expression revealed that USP19 expression was decreased in the acute MI group (acute MI: 0.12 ± 0.01, 3 d control: 0.31 ± 0.06, *p* < 0.01) and the CHF group (CHF: 0.15 ± 0.01, 10 w control: 0.35 ± 0.05, *p* < 0.01) (Figure 3A). However, none of the other DUBs, namely A20, USP14, UCH-L1 and CYLD showed a significant change in the mRNA expression in the acute MI group (A20: *p* = 0.52, USP14: *p* = 0.20, UCH-L1: *p* = 0.27, CYLD: *p* = 0.27) or at CHF (A20: *p* = 0.84, USP14: *p* = 0.08, UCH-L1: *p* = 0.08, CYLD: *p* = 0.06) as shown in Figure 3B–E.

### 2.5. Analysis of the Proteasome Activity

**Figure 4:**
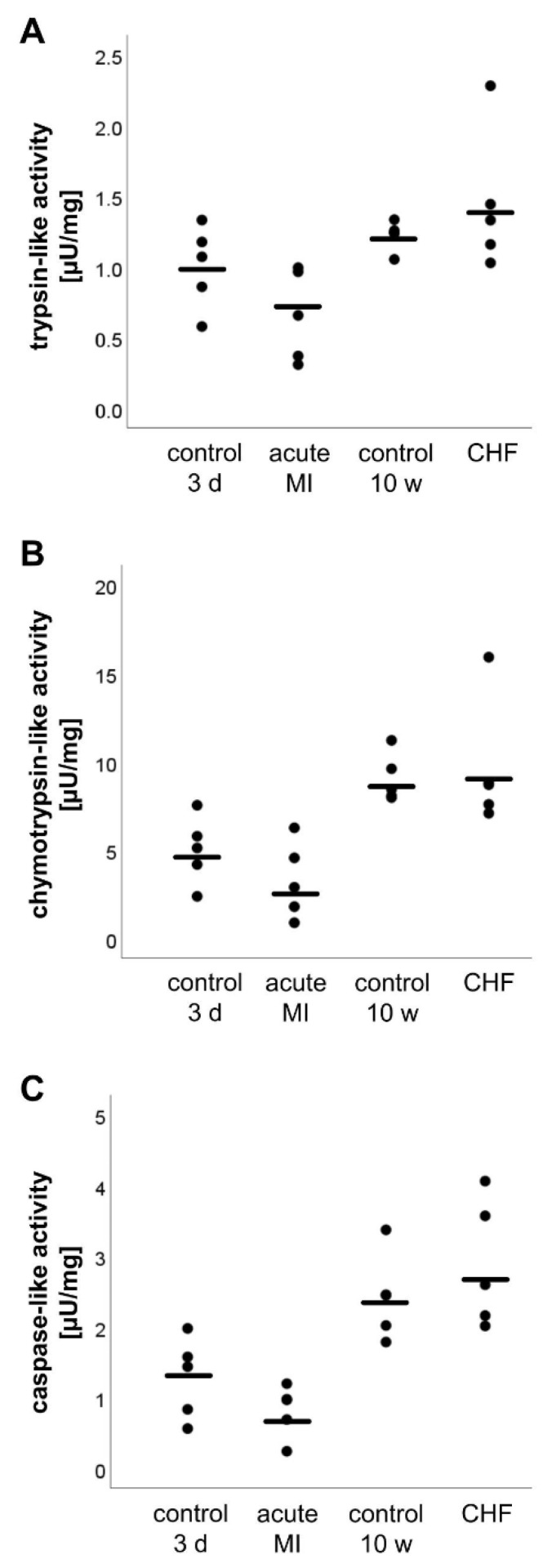
Proteasom activity of the trypsin- (**A**), chymotrypsin- (**B**) and caspase-like catalytic sites (**C**). Statistically significant results (*p* ≤ 0.05) were indicated.

Proteasomal activity was analyzed for the trypsin-, chymotrypsin-, and caspase-like catalytic sites of the proteasome. A change in the proteasomal activity was not detected for any of the aforementioned catalytic sites in both the acute MI group (trypsin-like activity: *p* = 0.12, chymotrypsin-like activity: *p* = 0.22, caspase-like activity: *p* = 0.17) and the CHF group (trypsin-like activity: *p* = 0.35, chymotrypsin-like activity: *p* = 0.75, caspase-like activity: *p* = 0.37) (Figure 4).

## 3. Discussion

In this study we reported the differential regulation of cardiac muscle-specific E3 ligases and DUBs following MI and CHF in a rodent model. Although the gene expression analysis following MI showed a downregulation of the E3 ligases MURF1, MAFBX, CHIP and MDM2, the protein expression was solely reduced for ITCH and MDM2. Our results reinforce the regulatory roles of ITCH and MDM2 in CHF, because these E3 ligases showed substantial changes in mRNA and protein expression. Furthermore, the gene expression of USP19 decreased following MI and CHF, whereas the gene expression of USP14, A20, UCH-L1 and CYLD was unaffected.

The potential of E3 ligases as therapeutic targets in cardiac diseases has been discussed previously [1,3,8]. However, the role of E3 ligases in the pathophysiology of MI and the structural and functional remodeling of the heart in patients with cardiomyopathy is not completely understood. There is a consensus that a defective control of proteostasis is implicated in cardiomyopathies, either as accumulation of incorrectly folded proteins or as acquired dysfunction of the protein quality control system [9]. MAFBX and MURF1 are muscle-specific E3 ligases that have been investigated extensively. Although MURF1 maintains the balance between hypertrophic and anti-hypertrophic signaling via the inhibition of the protein kinase C-mediated signaling in myocytes, MAFBX inhibits cardiac hypertrophy, prevents ischemic injury, and regulates the apoptosis of cardiomyocytes by polyubiquitination and degradation of calcineurin A [1,10]. We found that the mRNA expression levels of these two E3 ligases were decreased; however, no changes in the protein expression levels was observed in the mouse model following MI.

Previous reports on the quantifications of MAFBX and MURF1 following MI are contrary. The majority of studies analysing cardiac tissue observed that the expression of MURF1 and MAFBX was upregulated following CHF [11,12,13,14]. In contrast, a reduced mRNA expression of MAFBX and MURF1 has been observed in diseases accompanied by severe skeletal muscle atrophy and in MI, and it was hypothesized that an internal mechanism exists that aims at reducing further loss of muscle protein [15,16]. Our data are in accordance with these observations. Additionally, a considerable decrease in the MURF1 mRNA was observed in CHF. Furthermore, Spänig et al. found comparable results of MURF1 and MAFBX protein expression in the cardiac tissue obtained from patients of end-stage CHF caused due ischemic cardiomyopathy [17].

The E3 ligase ITCH controls the degree of thioredoxin (Trx)-interacting protein (TXNIP) degradation by the proteasome [18]. TXNIP is a part of the Trx system and prevents reactive oxygen species scavenging by Trx1 through its inhibition. In accordance with the results obtained from the study performed on human cardiac tissue of patients with ischemic cardiomyopathy [19], the mRNA and protein expression of ITCH was reduced following CHF in the murine model. We hypothesized that the downregulation of ITCH occurred during cardiac remodeling and not as a direct effect of the hypoxic conditions caused due to MI because we found stable mRNA and protein expression of ITCH 3 d after MI.

The E3 ligases CHIP and MDM2 regulate the degradation of p53 and the subsequently p53-mediated apoptosis [20,21]. Thus, the changes in the expression of both E3 ligases can influence the severity of diseases such as ischemia/reperfusion injury or MI [5]. In addition to the regulation of p53 activity, MDM2 interacts with the hypoxia-inducible factor (HIF)-1α, thereby enhancing the transcriptional activity of pro-angiogenic genes [22], and regulates forkhead box O transcription factors thereby influencing the antioxidant defense [23]. In our mouse model, CHIP and MDM2 mRNA expression decreased at 3 d after MI and remained at a low level during CHF. This decrease in CHIP and MDM2 expression was partially caused by HIF-1α and led to increased p53 expression [24,25]. An increase in p53 promotes apoptosis, thereby increasing the severity of MI-induced cardiac damage. Thus, we conclude that the downregulation of CHIP and MDM2 mRNA expression promotes p53-dependent apoptosis following MI. The increase in MDM2 protein expression after MI is contrary to the results obtained for mRNA expression. One possible reason could be the choice of the antibody used for MDM2 detection during Western blot analysis since the MDM2 protein exists in several isoforms and MDM2 is a component of different protein complexes such as TRIM28/KAP1-MDM2-p53/TP53 and TRIM28/KAP1-ERBB4-MDM2. Therefore, it is not clear which MDM2 isoforms have been detected by the antibody used. In the present study, the primers used for MDM2 mRNA analysis were designed for the detection of all splice variants and isoforms. Thus, it is possible to obtain contrary results in the mRNA and protein expression of MDM2. Furthermore, MDM2 expression increases with age [26], which could explain the remarkable increase in the 3 d and 10 w control groups after MI.

Moreover, DUBs play a critical role in cardiac-specific protein turnover. In this study, we investigated the changes in different DUB expression levels after MI. The mRNA expression of USP19 was reduced after MI and CHF whereas the expression of USP14, A20, UCH-L1 and CYLD was comparable with the control group. USP19 positively regulates autophagy by stabilizing Beclin-1, an essential autophagy protein, via deubiquitination [27]. Furthermore, USP19 negatively regulates type I interferon (IFN) signaling and the nuclear factor kappa-light-chain-enhancer of activated B cells (NF-κB) activation [27,28]. The reduced expression of USP19 following MI and CHF could result in reduced autophagic response and could promote IFN signaling and NF-κB activation induced by tumor necrosis factor or interleukin 1β. Moreover, the protein complex consisting of CHIP, BAG3, heat shock protein (Hsp) 40 and Hsp70 promotes protein degradation by autophagy [29]. Therefore, reduced expression of BAG3 and CHIP could result in a decreased rate of autophagy. In our study, decreased expression levels of BAG3 and CHIP after MI were observed, thereby promoting the hypothesis of a reduced autophagic response following MI. However, we did not quantify the autophagic response in the present study, and we did not detect an increase in proteasomal protein degradation after MI or CHF.

MDM2 can increase NF-κB activity [30], and the ITCH-CYLD protein complex terminates NF-κB activation via Tak1 [31]. In our study, increased MDM2 protein expression after MI and decreased ITCH expression after CHF could promote NF-kB activity, which in turn is known to induce heart failure by triggering chronic inflammation [32]. Hence, our hypothesis that NF-kB was activated after MI and CHF was confirmed by determining that MDM2 protein expression increased after MI and ITCH expression decreased following CHF. Furthermore, the E3 ligases ITCH and MDM2 interact with the ErbB4 protein that is known to activate mitochondrial apoptotic pathways [33]. Thus, changes in the expression of both E3 ligases could be responsible for an increase in mitochondrial apoptosis.

It has been demonstrated that the time point of E3 ligase quantification is important for understanding the role of different E3 ligases, based on the results of MDM2 showing that protein expression was increased following MI and decreased following CHF. The diverging results of different E3 ligases indicate a rapid change in E3 ligase expression during MI and CHF. Thus, a fast cellular switch between apoptosis inhibition and destruction of damaged tissue is hypothesized for the transition of MI to CHF. It is necessary to determine the optimal time point for the application of E3 ligase inhibitors regarding future therapies.

One further question is whether the downregulation in the mRNA expression of several E3 ligases after MI and in CHF is based on the changes of a putative context-specific upstream signaling pathway. One hypothesis is that the AKT/forkhead box O (FOXO) pathway could play an essential role in that process and leads the inhibition of E3 ligases to protect against a total protein breakdown. The hypothesis is supported by a study of [34].

One limitation of our study was the analysis of enzyme activity of the investigated E3 ligases and DUBs or whether the expression of both plays a role for the pathophysiology of MI or CHF because this study did not elucidate the molecular mechanisms. Both protein classes exert their function via enzymatic activity, but only relative protein quantification has been performed because of the absence of suitable detection methods.

Furthermore, the use of different antibodies for protein detection could be responsible for contrary results obtained in different studies since not all antibody manufacturers determine whether the antibodies can detect all isoforms of a protein. In addition, we did not measure purified proteasome activities. MG132 also inhibits other proteases, and this may induce a bias in the experimental setup. Furthermore, different studies used different reference genes for mRNA quantification; however, the stability of these reference genes vary between and within the species [35]. 

Future studies should also be focused on the detailed regulation of upstream and downstream cellular mechanisms. The modulation of the expression of E3 ligases, DUBS or proteasome activity could provide insight into future drug candidates.

## 4. Materials and Methods

### 4.1. Study Design 

**Figure 5:**
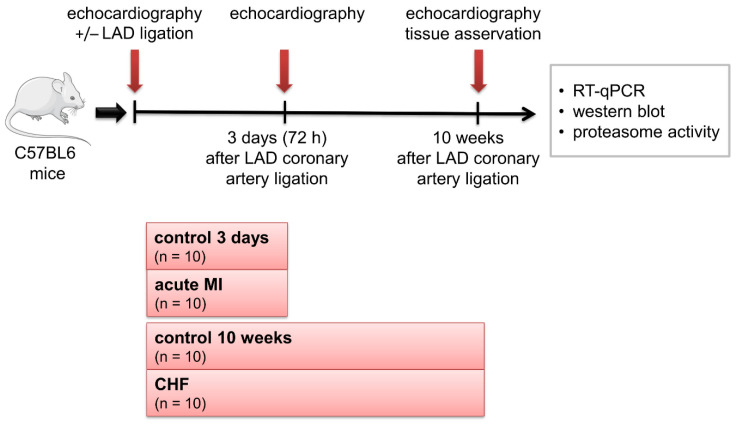
Study design. CHF, chronic heart failure; LAD, left anterior descending; MI, myocardial infarction; RT-qPCR, real-time quantitative polymerase chain reaction; 3 d, 3 days; 10 w, 10 weeks.

The animal study was approved by the local animal welfare agency (TVV 28/11) and conducted in accordance with 2010/63/EU. The study design is shown in Figure 5.

For inducing CHF, 12-week-old female mice (C57/BL6) were subjected to MI. Therefore, the ligation of the LAD coronary artery was performed as previously described [36]. Echocardiography was performed to confirm MI, and only mice with a large infarct (left ventricular ejection fraction (LVEF) < 20%) were subsequently used in this study.

The mice were euthanized 3 days (3 d) (acute MI group, *n* = 10) or 10 weeks (10 w) after the surgery (CHF group, *n* = 10), whereas sham animals that underwent surgery but where the LAD was not ligated served as control groups (*n* = 10 each) at each time point to collect tissues for functional and molecular characterization (Figure 5). 

### 4.2. Echocardiography Histology of the Heart 

Echocardiography was performed in M-mode at the start of the study, 3 days and 10 weeks post-surgery, as previously described [37]. In brief, the left ventricular fractional shortening (LVFS) was calculated by the assessment of LV end-diastolic (LVEDD) and systolic (LVESD) diameters (LVFS = [LVEDD − LVESD/LVEDD] × 100). After euthanasia, the base and apex of the heart were pooled, snap-frozen in liquid nitrogen and stored at −80 °C until further analysis. The medial portion of the heart was fixed in 4% phosphate-buffered saline (PBS)-buffered formalin and paraffin-embedded for immunohistological analysis. Serial cross sections (2µm) were subsequently HE-stained for further analysis. A computer imaging software (Analysis 3.0, Olmpus Soft Imaging Solutions GmbH, Münster, Germany) was used to define the infarct boundary, specified by a significant loss in the LV myocardium (i.e., a thinning in the LV wall ± 2 SCs of mean wall thickness), as previously described [7].

Average infarct size (%) was then quantified as the ratio of the infract circumference-to-overall LV circumference.

### 4.3. RNA Isolation

RNA was isolated from ≤30 mg of snap-frozen myocardial tissue using the RNeasy^®^ Fibrous Tissue Mini Kit (Qiagen GmbH, Hilden, Germany) according to the manufacturer’s instructions. The concentration and purity of RNA were measured at the BioPhotometer (Eppendorf, Hamburg, Germany).

For reverse transcription, the QuantiNova™ Reverse Transcription Kit (Qiagen) was used according to the manufacturer’s instructions. In brief, 6 µL (2 µg) RNA, 2 µL gDNA removal mix, and 7 µL RNase-free water were mixed and incubated for 2 min at 45 °C following addition of 4 µL reverse transcription mix and 1 µL Reverse Transcription enzyme. The mixture was incubated for 3 min at 25 °C, 10 min at 45 °C, 5 min at 85 °C, and cooled down to 4 °C. The transcribed cDNA was stored at −20 °C for reverse transcription quantitative polymerase chain reaction (RT-qPCR) analysis.

### 4.4. RT-qPCR Analysis

For RT-qPCR analysis, QuantiNova™ SYBR^®^ Green PCR Kit (Qiagen), ≤100 ng of cDNA, and 5 μM of forward and reverse primers were used according to the manufacturer’s instructions. EditSeq software (DNASTAR^®^, Madison, WI, USA) and Primer 3 software version 4.1.0 [38] were used to design oligonucleotide primers that flank intron sequences of the target gene if possible. The forward and reverse primers for the following genes were used to perform RT-qPCR: MURF1, MAFBX, ITCH, CHIP, MDM2, USP19, USP14, A20, UCH-L1, CYLD, BAG3 and glyceraldehyde 3-phosphate dehydrogenase (GAPDH) The primer sequences were shown in Table 2. RT-qPCR was performed using a LightCycler^®^ 480 (Roche Diagnostics, Mannheim, Germany) and LightCycler^®^ 480 software 1.5.0 SP3 with the following protocol: 2 min at 95 °C, 40 cycles of 5 s at 95 °C and 10 s at 60 °C. Subsequently, the melting curves were recorded, and the correct size of the amplicons was determined by agarose gel electrophoresis. Threshold cycle (C_T_) values were set within the exponential phase of the RT-qPCR. Data were normalized to GAPDH expression, and 2^−ΔΔCT^ values were used to calculate the relative expression levels of the genes.

### 4.5. Proteasomal Activity

The snap-frozen myocardial tissue samples were homogenized in a relaxing buffer (90 mM HEPES, 126 mM KCl, 36 mM NaCl, 1 mM MgCl, 50 mM EGTA, 8 mM ATP, 10 mM creatine phosphate) containing 1× protease and phosphatase inhibitor cocktail (Thermo Scientific, Waltham, MA, USA), and sonicated in chilled water using an ultrasound device (Bandelin, Berlin, Germany). Afterwards, the protein content of the cytosolic fraction was determined using the bicinchoninic acid assay (Thermo Fisher Scientific, Waltham, MA, USA) following the manufacturer’s specifications.

Chymotrypsin-, trypsin-, and caspase-like proteasomal activities were measured using fluorogenic peptides, namely, Suc-LLVY-AMC (Sigma Aldrich, St. Louis, MO, USA), Bz-VGR-AMC (Enzo Life Sciences, Exeter, UK) and Z-LLG-AMC (Sigma Aldrich). The cytosolic proteins (20 µg) were incubated with reaction buffer (50 mM Tris, 0.5 mM EDTA) and 4 mM of each fluorogenic peptide at a time. Subsequently, the reaction was inhibited by adding 20 µM of proteasome inhibitor MG132 (Sigma Aldrich) which served as a negative control. The kinetic reaction was measured using a spectrophotometer (Tecan, Crailsheim, Germany) for 10 min at 380 nm excitatory and 440 nm emission wavelengths. For the calculation of the enzymatic activity, a calibration curve of free 7-amino-4-methylcoumarine was generated (Sigma Aldrich).

### 4.6. Immunoblot/Sodium Dodecyl Sulfate-Polyacrylamide Gel Electrophoresis and Western Blotting Analysis

The snap-frozen tissue samples were homogenized in lysis buffer (50 mM Tris, 1 mM EDTA, 150 mM NaCl, 1% Nonidet-P40, 0.25% sodium deoxycholic acid, 1× protease and phosphatase inhibitor cocktail (Thermo Fisher Scientific), and 1% phenylmethylsulfonylfluoride), sonicated in chilled water using an ultrasound device and centrifuged at 14,000× *g* for 10 min at 4 °C. The protein content was determined using the bicinchoninic acid assay. The proteins were separated using sodium dodecyl sulfate-polyacrylamide gel electrophoresis (SDS-PAGE) and transferred to a polyvinylidene fluoride membrane by electrophoretic transfer with Towbin transfer buffer (25 mM Tris, 0.7 mM SDS, 192 mM glycine, 20% (*v*/*v*) methanol) as previously described [39]. Blotting membranes were blocked with Roti^®^-Block (Roth, Karlsruhe, Germany) for 30 min at 21 °C followed by overnight incubation at 4 °C with primary antibodies for the E3 ligases MURF1, MAFBX, ITCH, CHIP and MDM2 (all purchased from Abcam, Cambridge, UK). After washing with Tris-buffered saline-Tween 20 (TBS-T; 50 mM Tris, 0.5 M NaCl, 1% Tween 20), the blotting membrane was incubated with either horseradish peroxidase (HRP)-conjugated secondary antibodies (Sigma Aldrich) or biotinylated HRP-labeled streptavidin antibodies (Sigma Aldrich and Thermo Fisher Scientific) for 1 h at 21 °C. GAPDH (Hytest, Turku, Finland) was used as an internal standard. For this purpose, the blotting membrane was incubated with GAPDH-antibody overnight at 4 °C. The next day, the membrane was washed with TBS-T following incubation with the appropriate HRP-conjugated secondary antibodies (Sigma Aldrich). Subsequently, the signals were detected using a chemiluminescence-based detection system (WesternBright Quantum chemiluminescence substrate; Biozym Scientific, Hessisch Oldendorf, Germany). The proteins were quantified by densitometry using the Fusion VisionCapt software version 16.13 (VWR, Radnor, PA, USA). The results are expressed as the ratio of the intensities of the samples and the internal standard.

### 4.7. Statistical Analysis

Statistical analysis was performed using SPSS Statistics 25 software (IBM, New York, NY, USA). Unless stated otherwise, the data are represented as mean ± standard deviation. We compared the means of two groups using unpaired two-sided Student’s *t*-test. For all analyses, a *p*-value ≤ 0.05 was considered as statistically significant.

## 5. Conclusions

We detected differential regulation of myocardial E3 ligases and the DUB USP19 in ischemic heart failure. The simultaneous downregulation in the mRNA expression of several E3 ligases following MI raises the question of whether an upstream regulation exists that is activated by MI for tissue protection or whether the downregulation of E3 ligases enables myocardial hypertrophy following MI. Additionally, the regulatory roles and suitability of ITCH, MDM2 and USP19 as therapeutic targets, as well as the role of NF-κB activation in CHF, should be clarified in future studies.

## Figures and Tables

**Table 1:** Characteristics of the study groups at the end of the evaluation period. Evaluation period ended 3 days (3 d) after LAD coronary artery ligation for the groups control 3 d and acute MI or 10 weeks (w) after LAD coronary artery ligation for the groups control 10 w and CHF.

	Control 3 d (*n* = 10)	Acute MI (*n* = 10)	*p* Value	Control 10 w (*n* = 10)	CHF (*n* = 10)	*p* Value
Body weight [g]	18.5 ± 0.7	16.4 ± 0.9	<0.01	22.0 ± 1.0	21.9 ± 1.9	0.90
Heart weight [mg]	94.1 ± 7.9	116.9 ± 16.6	<0.01	111.9 ± 15.8	175.3 ± 67.8	<0.01
Ejection fraction [%]	60.5 ± 11.9	17.2 ± 17.6	<0.01	59.3 ± 14.6	15.0 ± 4.5	<0.01
Fractional shortening [%]	31.9 ± 8.1	11.5 ± 6.6	<0.01	31.9 ± 10.7	6.8 ± 2.0	<0.01
Infarct size [%]	0.0 ± 0.0	57.1 ± 3.3	<0.01	0.0 ± 0.0	42.5 ± 13.1	<0.01

CHF, chronic heart failure; LAD, left anterior descending; MI, myocardial infarction; 3 d, 3 days; 10 w, 10 weeks.

**Table 2:** Primer sequences and RT-qPCR product length.

Gene		Sequence (5′-3′)	RT-qPCR Product Lenght (bp)
MURF1	forward	tgagtaactgcatctccatgct	118
	reverse	tcacctggtggctattctcc	
MAFBX	forward	tttgcaaacactgccacatt	106
	reverse	cttgaggggaaagtgagacg	
ITCH	forward	gggaggatttgctgacctta	121
	reverse	tccaggcggttaaaacaagt	
CHIP	forward	ccagctggagatggagagtt	122
	reverse	gcgaagggcactaggaatatc	
MDM2	forward	taatctccgcttggaaggac	104
	reverse	tctgtagcccttgatgaggaa	
USP19	forward	tatcgaaggttgtcccttgc	111
	reverse	gcgtgtgggtattcacagatt	
USP14	forward	cccagtgagtaagtccttagatgtt	100
	reverse	tcaaatcagaccagatcacga	
A20	forward	cacgactcacctgatcaacg	105
	reverse	cgtgctgaacaagctcaaag	
UCH-L1	forward	gaccatcggaaactcctgtg	134
	reverse	ggacagcttctccgtttcag	
CYLD	forward	acgagtgcagggagtgctat	116
	reverse	ttcaacctcctgggatgaag	
BAG3	forward	cagtggttgacaggcctca	100
	reverse	ctgggcctggcttactttct	
GAPDH	forward	aactttggcattgtggaagg	132
	reverse	ggatgcagggatgatgttct	

A20, TNF-α-induced protein 3; BAG3, Bcl-2-associated athanogene 3; bp, base pair(s); CHIP, carboxyl-terminus of Hsc70 interacting protein; CYLD, CYLD lysine 63 deubiquitinase; GAPDH, glyceraldehyde 3-phosphate dehydrogenase; ITCH, E3 ubiquitin-protein ligase Itchy homolog; MAFBX, muscle atrophy F-box; MDM2, mouse double minute 2 homolog; MURF1, muscle ring finger 1; RT-qPCR, real time quntitative polymerase chain reaction; UCH-L1, ubiquitin carboxy-terminal hydrolase L1; USP14/19, ubiquitin carboxyl-terminal hydrolse 14/19.

## Data Availability

The authors confirm that the data supporting the findings of this study are available within the article and/or its supplementary materials.

## Supplementary Materials

The following are available online at https://www.mdpi.com/article/10.3390/life11121430/s1, Figure S1: Protein expression of the E3 ligases MURF1 (A), MAFBX (B), ITCH (C), CHIP (D) and MDM2 (E) by Western blotting.
