# Supplementary material for: Differential Regulation of Myocardial E3 Ligases and Deubiquitinases in Ischemic Heart Failure
Source: Life (Basel). 2021 Dec 18;11(12):1430. doi: 10.3390/life11121430 (PMC8708923; doi:10.3390/life11121430)
Supplement: Supplementary file 1 [file life-11-01430-s001.zip › life-1484140-supplementary.pdf]

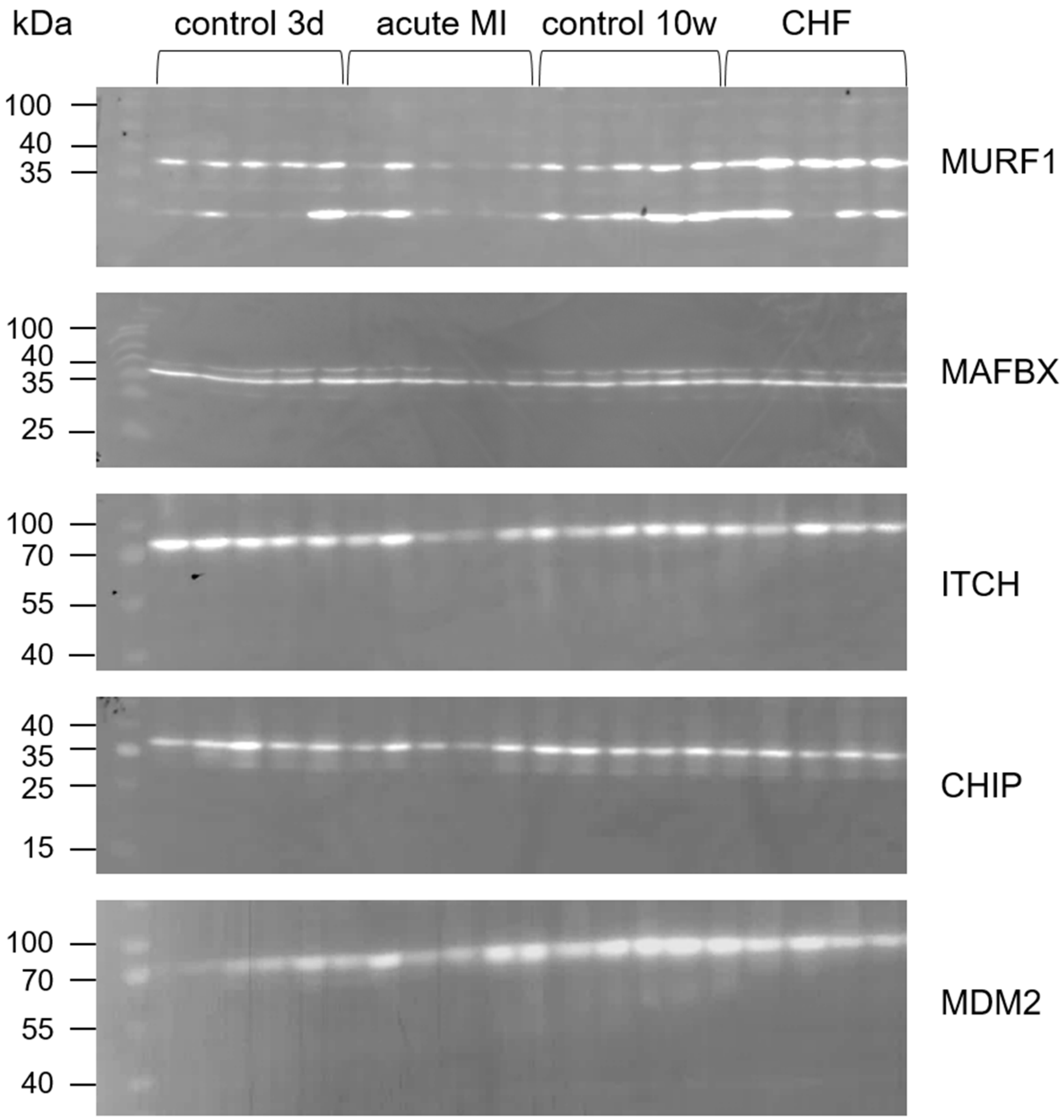


**Figure S1.** Protein expression of the E3 ligases MURF1 (A), MAFBX (B), ITCH (C), CHIP (D) and MDM2 (E) by immunoblotting. Protein expression was determined by western blot analysis and normalized to GAPDH protein expression. CHF, chronic heart failure; CHIP, carboxyl-terminus of Hsc70 interacting protein; ITCH, E3 ubiquitin-protein ligase Itchy homolog; kDA, kilodalton; MAFBX, muscle atrophy F-box; MDM2, mouse double minute 2 homolog; MI, myocardial infarction; MURF1, muscle ring finger 1; 3d, 3 days; 10w, 10 weeks.
